# Volatile Metabolites

**DOI:** 10.3390/metabo1010041

**Published:** 2011-11-25

**Authors:** Daryl D. Rowan

**Affiliations:** The New Zealand Institute for Plant and Food Research Limited, Private Bag 11600, Palmerston North 4442, New Zealand; E-Mail: daryl.rowan@plantandfood.co.nz; Tel.: +64-6-953-7685; Fax: +64-6-953-7702

**Keywords:** metabolomics, volatiles, volatile profiling, aroma, flavor, semiochemicals, gas chromatography, mass spectrometry

## Abstract

Volatile organic compounds (volatiles) comprise a chemically diverse class of low molecular weight organic compounds having an appreciable vapor pressure under ambient conditions. Volatiles produced by plants attract pollinators and seed dispersers, and provide defense against pests and pathogens. For insects, volatiles may act as pheromones directing social behavior or as cues for finding hosts or prey. For humans, volatiles are important as flavorants and as possible disease biomarkers. The marine environment is also a major source of halogenated and sulfur-containing volatiles which participate in the global cycling of these elements. While volatile analysis commonly measures a rather restricted set of analytes, the diverse and extreme physical properties of volatiles provide unique analytical challenges. Volatiles constitute only a small proportion of the total number of metabolites produced by living organisms, however, because of their roles as signaling molecules (semiochemicals) both within and between organisms, accurately measuring and determining the roles of these compounds is crucial to an integrated understanding of living systems. This review summarizes recent developments in volatile research from a metabolomics perspective with a focus on the role of recent technical innovation in developing new areas of volatile research and expanding the range of ecological interactions which may be mediated by volatile organic metabolites.

## 1. Introduction

**Figure 1.**
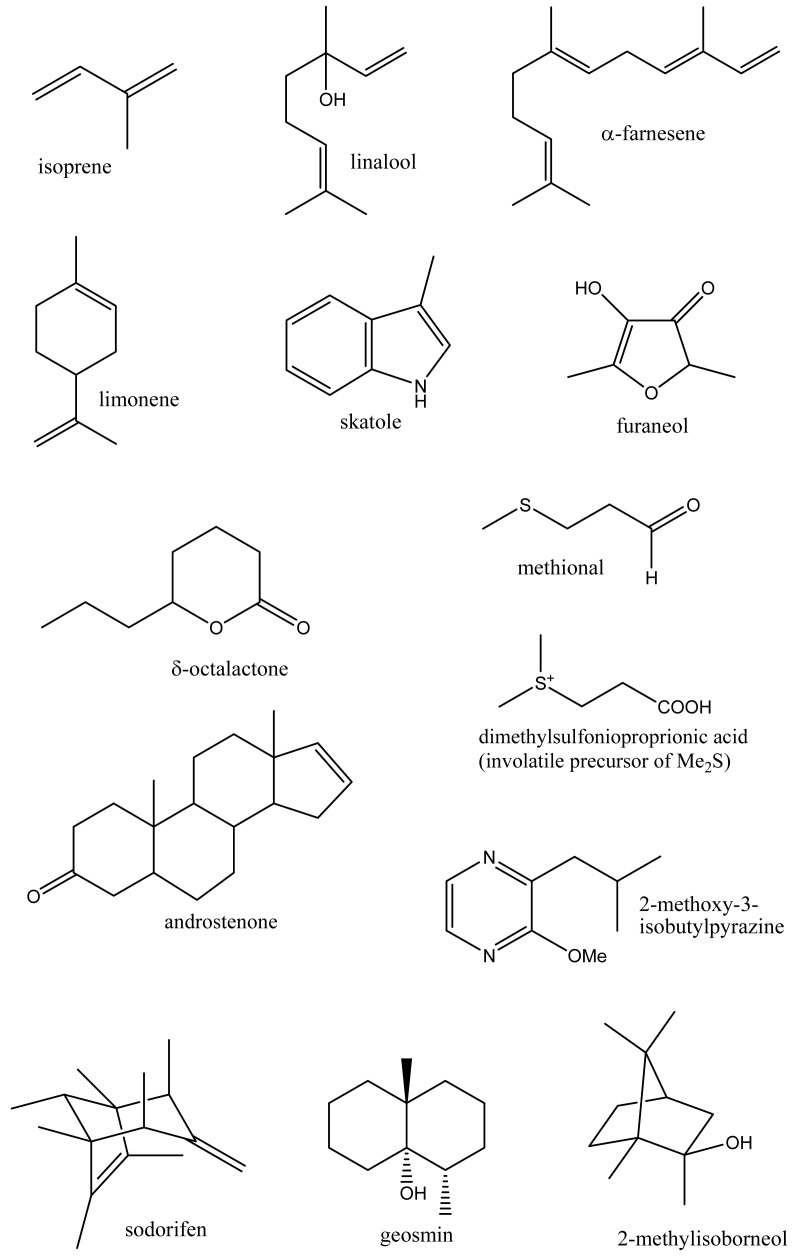
Chemical structures of some plant, microbial and mammalian derived volatiles.

Volatile organic compounds (volatiles) comprise a chemically diverse group of organic compounds, generally with a molecular weight in the range of 50–200 Daltons and having appreciable vapor pressure under ambient conditions. Thousands of different volatile compounds occur in nature including both naturally occurring compounds and those produced as a result of human activities. These compounds occur at a wide range of concentrations and include ubiquitous hydrocarbons and aromatic compounds such as the xylenes and monoterpenes such as α-pinene, to trace amounts of insect pheromones and important flavor compounds detectable by humans at below part per trillion (ppt) levels. Their high vapor pressure and low molecular weight means volatiles can readily diffuse through the gas phase and within biological systems and hence can serve as signaling molecules (semiochemicals) passing information both within and between organisms, functioning as hormones or in the identification of food, mates, co-specifics, competitors, predators or suitable habitat [1,2]. Volatiles arise by a variety of biosynthetic routes but principally from amino and fatty acids, and terpene biosynthetic pathways [1] and include a wide range of chemical classes (hydrocarbons, aromatics, alcohols, aldehydes, acids, esters, amines and thiols) with a range of physical properties from gases at room temperature (ethylene, Table 1) to higher molecular weight compounds such as skatole and androstenone (Figure 1) which possess sufficient vapor pressure and biological activity to be clearly perceived by humans [3].

For humans and other animals, volatiles are important as scents and contribute to the flavor of food (flavor volatiles). Volatiles, as food aromas, contribute to the palatability and to our appreciation of foods, and along with sugars, organic acids, salts and other components affecting taste receptors, are responsible for the food flavor. For plants, volatiles contribute to the attraction of insects to pollinate flowers and advertise that fruit are ripe and ready for seed dispersal. Plants emit volatiles from their roots, leaves, fruits and flowers, and use these compounds internally as defensive signaling systems to modulate levels of systemic acquired resistance (SAR) to pests and diseases [4-6] and to alleviate heat and oxidative stress (isoprene) [2,7,8]. Plant volatiles include phytohormones such as ethylene, and methyl salicylate and jasmonate which are used to communicate the presence of herbivores to neighboring plants [5,9]. Plant volatiles are also involved in beneficial tritrophic interactions where the action of insect feeding on a plant results in the release of volatiles which attract predatory insects [2,5,9-11]. Volatile production in living systems shows complex and dynamic interactions with the environment including short and long time span responses to light, stress, pollination or predation [5,12].

**Table 1.** A selection of volatile metabolites arranged in order of increasing boiling point and chosen to illustrate the different physical and chemical properties of this class of metabolite.

**Volatile**	**Molecular formula**	**Molecular weight**	**bp** °**C (760 mmHg)**	**Chemical class / biosynthesis**	**Source / bioactivity**
ethylene	C_2_H_4_	28	−102	Hydrocarbon from methionine	Plant hormone with roles in fruit ripening, senescence and stress responses
methanethiol	CH_3_SH	48	6	sulfide	biogenic, odor of rotten cabbage
isoprene	C_5_H_8_	68	34	terpenoid	oxidative stress response of plants
ethanol	C_2_H_5_OH	46	78	alcohol	anaerobic respiration
dimethyl disulfide	CH_3_SSCH_3_	94	109	disulfide	onion, garlic, coffee flavoring
hexanal	C_6_H_12_O	100	131	aldehyde via lipoxygenase	green leaf volatile, green, grassy odor
ethyl 2-methybutanoate	C_7_H_14_O_2_	130	133	ester from isoleucine	key flavor compound of fruit
3-*Z*-hexenol	C_6_H_12_O	100	156	alcohol via lipoxygenase	green leaf volatile, herbaceous odor
methional	C_4_H_8_S_1_O_1_	104	165	thioether aldehyde	flavor compound
linalool	C_10_H_18_O	154	198	monoterpene alcohol	common plant volatile with floral odor
2*Z*,6*E*-nonadienal	C_9_H_14_O_1_	138	203	aldehyde via lipoxygenase	cucumber flavor
furaneol^®^	C_6_H_8_O_3_	128	216	sugar derived	strawberry furanone, meaty flavoring agent
δ-octalactone	C_8_H_14_O_2_	142	238	fatty acid oxidation	microbial, dairy foods—Coconut, creamy, fruity
hexyl hexanoate	C_12_H_24_O_2_	200	245	ester from fatty acid degradation	fruity ester
skatole	C_9_H_9_N_1_	131	265	Aromatic heterocycle	tryptophan, Faecal, fruity odor at low levels
α-farnesene	C_15_H_24_	204	280	sesquiterpene	flower volatile and flavor precursor
androstenone	C_18_H_28_O_1_	272	372	steroid	mammalian pheromone

**Figure 2.**
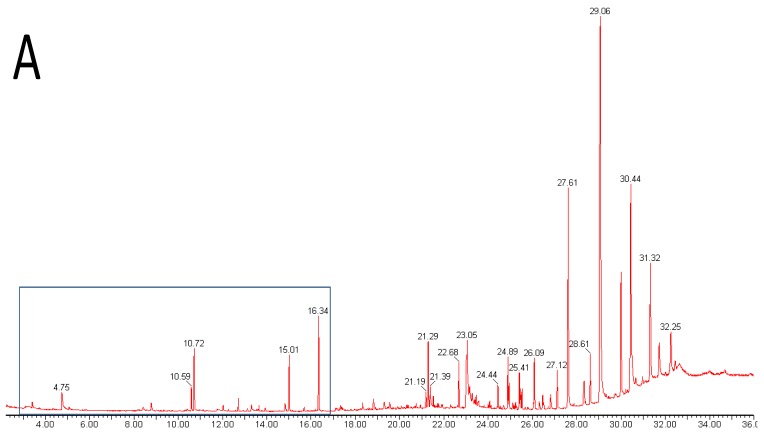

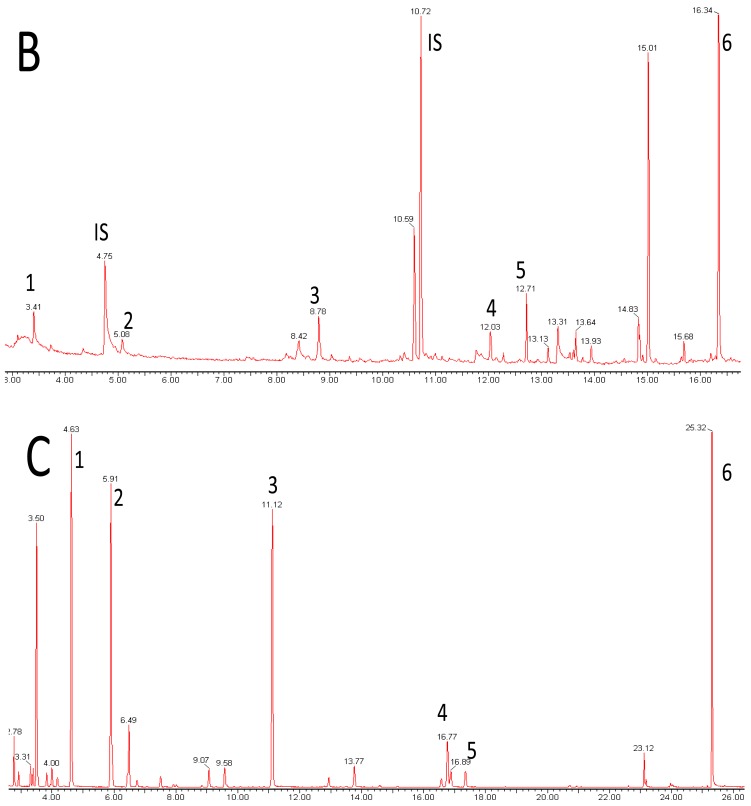
GC-MS (TIC) chromatograms of apple fruit volatiles showing the dependency of the volatile profile upon the methodology used, and the chemical simplicity and selectivity of a typical headspace profile. A complete chromatogram of the diethyl ether extract of Jazz™ (‘SciFresh’) apple skin (**A**) showing the volatiles in the region (3–16 min), and an expansion of this volatile region (**B**), is compared with the headspace volatile profile obtained from a ‘Royal Gala’ x ‘Granny Smith’ apple (**C**). Chromatographic separations used DB5 and Carbowax GC columns to ensure the elution of high boiling components and to maximize isomeric separations respectively. Labeled peaks are butyl acetate (1), 2-methylbutyl acetate (2), hexyl acetate (3), butyl hexanoate (4), hexyl 2-methylbutanoate (5), α-farnesene (6) and internal standard (IS).

Metabolomics is metabolite analysis applied with the aim of providing a comprehensive analysis of both the concentrations of all the metabolites present in a biological sample and an understanding of their significance within some larger biological context [15-19]. This goal of achieving both a comprehensive measurement of metabolite concentrations and knowledge of biological responses is limited by the chemical diversity of the metabolites present in biological samples and limitations of the current analytical methods. Volatile analysis sits somewhere between an idealized comprehensive metabolomics and traditional targeted chemical analysis. The restricted number of metabolites generally considered in volatile samples eases problems of automated metabolite detection and identification and of data analysis (the samples are simpler and chemically less ‘noisy’). However, the vastly different physical properties of volatile metabolites, including biologically important gases such as ethylene, isoprene and nitric oxide (NO), lipophilic hydrocarbons, esters, terpenes and sterols, and highly polar, water soluble flavor compounds, such as methionol (3-methylthiopropanol) and furaneol^®^ (Table 1 and Figure 1), limit the range of volatiles that can be measured by any particular method. As a corollary, those compounds which are detected and measured will be highly dependent on the volatile sampling method used. Figure 2 shows the differences in GC-MS chromatograms of volatiles recovered from apple skin using either solvent extraction or headspace trapping to collect the volatile metabolites. Headspace sampling results in a simpler and more selective volatile profile with a pronounced bias towards the butyl, 2-methylbutyl and hexyl acetate esters important for apple aroma [20] whereas extraction with organic solvents also recovers higher molecular weight terpenes and hydrocarbons which dominate the chromatogram.

This review summarizes recent developments in volatile research from a metabolomics perspective with a focus on the role of recent technical innovations in developing new areas of volatile research and expanding the range of ecological interactions which may be mediated by volatile organic metabolites. Volatile analysis measures a restricted set of analytes but has a long history in sensory and food analysis of combining chemical and biological data in multivariate statistical models. Future directions of volatile research are discussed.

## 2. Methodology for Volatile Analysis

As with metabolomics, the measurement of the profile of volatiles produced by a living system requires a shift from the selective quantitative analysis of specific metabolites, which has been the focus of analytical chemistry, to the measurement of multiple metabolites with corresponding tradeoffs in the precision, accuracy and sensitivity with which individual metabolites can be measured. The diverse and ‘extreme’ physical properties of some volatiles provide unique challenges and instructional examples for metabolomics analysis. As the various metabolites within a volatile profile will differ markedly in their physical and chemical properties (Table 1 and Figure 1), the efficiency with which we can extract these volatiles from the sample will also differ widely and will be highly dependent on the sampling methods used. Thus, volatile profiles are highly method-dependent and the match of sampling methodology and sample handling with the chemical properties of the volatiles present in the sample will frequently determine which members of the complete volatile profile will be detected. Consequently, and in common with metabolomics, no single analytical technique can give a complete profile of all volatiles, and it appears that a combination of broad-spectrum profiling methods, and of targeted methods to analyze key volatiles that may occur at very low concentrations (e.g., hormones, key flavor impact compounds), will continue to be used. While a variety of methods can be used to collect and concentrate volatile metabolites from a sample, there are two basic approaches: direct sampling of volatiles from the air (headspace) surrounding a sample or solvent extraction of the volatiles from tissue or environmental samples with subsequent purification to remove non-volatile materials which may interfere with subsequent instrumental analysis.

### 2.1. Headspace Methods for Volatile Collection

Dynamic (purge and trap) headspace sampling and static headspace sampling are two methods widely used to collect or concentrate volatiles from the air surrounding a sample (the headspace) [21]. In static headspace analysis, the volatiles in the sample are allowed to equilibrate with the air in an air-tight container. After equilibration, a known volume of air is collected from above the sample, usually in a gas-tight syringe, and injected directly into the gas chromatograph. In dynamic (purge and trap) headspace sampling, a known volume of purified air is passed over the sample and the entrained volatiles are concentrated onto an adsorbent trapping material (graphite or an organic polymer such as Tenax). In a variant of this technique, closed-loop stripping, the air flow is recycled through the adsorbent trap. Volatiles may then be removed from the adsorbent trap by elution with organic solvents (commonly with diethyl ether) or transferred directly to the gas chromatograph (GC) by rapid heating of the adsorbent material in a stream of inert gas. This latter approach (thermal desorption) transfers all volatiles collected on the adsorbent trap onto the GC column giving much greater sensitivity and the ability to observe the more volatile metabolites that would otherwise be obscured on injection in an organic solvent. Headspace methods to sample volatile emission from a variety of biological samples including from skin, plant leaves, and in breath and rumen gases have been reviewed [22,23]. Detailed protocols for the headspace sampling of plant volatiles are reviewed by Qualley and Dudareva [21].

Alternatively, solid-phase micro extraction (SPME) may be used to pre-concentrate volatiles from the headspace, or from solution, prior to analysis. In this technique, the adsorbent is coated onto a glass fiber held within a hypodermic needle which protects the adsorbent during passage through the septa of sampling ports and GC injectors. For volatile analysis, the adsorbent-coated fiber is extended from the protecting needle and exposed to volatiles in the headspace above a sample. Volatiles adsorb onto the fiber, which after some fixed time is removed and inserted into the heated injection port of a gas chromatograph. Volatiles are desorbed off the fiber in the heated injector (thermal desorption) and are carried onto the GC column. SPME (and purge and trap thermal desorption) sampling of volatiles usually occurs as an integrated process in real time although SPME fibers and desorption traps may be stored at low temperatures. Adsorbed volatiles may be measured only once and this can be a risky and stressful approach when each sample represents an irreplaceable snapshot of the system under study.

Quantitation with SPME can be difficult when analytes compete for binding sites on the SPME fiber or where the equilibration of volatiles with the absorbent on the fiber follows complex kinetics or is slow relative to volatile sampling times [23,24]. Microwave assisted extraction (MAE), using microwave heating of adsorbed water in the sample, is another approach to assist in obtaining more rapid equilibration and more complete extraction of volatiles from complex matrices. MAE has been optimized to determine volatile profiles from powdered dry stems and leaves of the Chinese medicinal herb *Gymnotheca involucrata* by GC-MS SPME with RSDs of less than 9% [25]. Magnetic stirring bars coated with a volatile adsorbing phase (Twister™, Gerstal GmbH) may also be used to concentrate volatiles from more demanding and complex matrices such as human saliva [26] or to distinguish between urinary volatile profiles of mice [27]. Volatiles are subsequently thermally desorbed from the surface of the stir bars and analyzed by GC-MS. Recently biocompatible coatings for SPME fibers have been developed which allow *in vivo* sampling of blood and other complex fluids [23,28].

### 2.2. Solvent Based Volatile Extraction Methods

Extraction with organic solvents generally gives a more complete profile of volatile metabolites including representation from polar hydrophilic species such as the lower molecular weight alcohols, hydroxyl-acids, thiols, and flavor compounds such as acetoin, methionol and furaneol^®^ (Figure 1). However, non-volatile material such as leaf waxes, triterpenes, sterols, triglycerides and more complex lipids, and silicones and plasticizers from laboratory apparatus, are also likely to be extracted (Figure 2) and may complicate analysis unless removed or the analytical method is suitably modified. Solvents chosen to optimize the profile of extracted metabolites include pentane—ether mixtures and dichloromethane. Unwanted interfering compounds such as lipids, pigments and hydrocarbons, may be removed by distillation (simultaneous distillation-extraction (SDE) [29], vacuum micro distillation or solvent assisted flavor evaporation (SAFE) [30], or by adsorption chromatography (solid phase extraction). Vacuum micro distillation, using liquid nitrogen to distil and condense organic extracts under vacuum, also appears a useful technique to isolate volatile fractions suitable for instrumental analysis from complex matrices such as urine and faeces [31]. Atmospheric pressure (SDE) and steam distillation (hydrodistillation) methods used to prepare volatile extracts for GC-MS analysis are liable to artifact formation due to the use of heat [29,32].

Solvent extracts are routinely concentrated by evaporation before analysis, increasing sensitivity but resulting in selective loss of the more volatile metabolites as a function of the extent of the volume reduction. These losses may be compensated for by the use of internal standards which are generally added during sample extraction and are used to correct for any loss of volatiles that occurs during the process of sample preparation. Internal standards are generally more easily used with solvent extraction than with headspace methods. Since only a small portion (1 μL) of the final solvent extract is usually used for GC-MS analysis, solvent extraction methods offer less sensitivity than direct thermal desorption or SPME. Solvent extracts, prepared either by solvent extraction or elution of headspace sampling adsorbents provide the most convenient method of sample handling. Samples can be easily stored before analysis, introduction into the GC is readily and reliably automated, and there is usually sufficient sample for multiple analyses facilitating robust identification and quantification of both known and unknown volatiles.

An alternative to the use of organic solvents is extraction with supercritical fluids (SCF) usually supercritical carbon dioxide, either pure or in the presence of chemical modifiers. Supercritical carbon dioxide has a polarity comparable to pentane and has been used to obtain volatiles and essential oils from a wide range of plant species [33]. While SCF extraction has the advantage of using a totally volatile solvent, specialized equipment is required. SCF extraction has been compared with conventional solvent and Soxhlet extraction, hydrodistillation, and simultaneous distillation-extraction (SDE) methods of volatile extraction [32,33].

### 2.3. Instrumentation for the Measurement of Volatiles

By far the most commonly employed instrumentation for profiling volatiles is gas chromatography mass spectrometry (GC-MS). GC coupled to detection by electron impact mass spectrometry (EI-MS) provides high chromatographic resolution, sensitivity, compound-specific detection, quantification, and the potential to identify unknown volatiles by characteristic and reproducible fragmentation spectra in addition to their retention times on the gas chromatograph. Sample analysis is simplified compared with silylation-based methods for the GC analysis of primary metabolites in that no chemical derivatization is required and the chromatograms generally contain fewer metabolites and less chemical noise (Figure 2). A variety of commercial and web-based resources can be used to identify unknown compounds in a given volatile sample including large databases of searchable mass spectral libraries. High-resolution time-of-flight GC-MS instruments enable highly accurate measurement of ion masses (*m*/*z* ratios). This allows the calculation of chemical formulae and aids the identification of unknown metabolites. The use of chemical detectors other than the mass spectrometer, sulfur selective detectors or the human nose in gas chromatography-olfactometry (sniffer port, GC-O), may enable more specific and sensitive detection of particular metabolites.

To improve the separation or ensure the comprehensive detection of all volatile components in complex or difficult volatile matrices, various multidimensional GC (MD-GC) methods have been developed. MD-GC methods use a liquid nitrogen or carbon dioxide cooled interface to trap volatiles eluting from one GC column before their introduction into a second GC column carrying a different stationary phase. The analysis of portions of the volatile profile on a second GC phase with a different selectivity allows an orthogonal separation of complex volatile samples and the detection of volatiles that are not detected on conventional one-dimensional GC. Thus repetitive ‘heart-cutting’ multidimensional GC-MS of lavender essential oil [34] more than tripled the number of peaks detected with decreased peak widths and improved peak capacity. The combination of MD-GC analysis with gas chromatography olfactometry (GC-O) allows exhaustive characterization of odor active volatiles in complex essential oils and food plants and the resolution of key odorants from co-eluting metabolites [35].

#### 2.3.1. Chromatography-Free Methods (PTR-MS/SIFT-MS/IMS)

A limitation of GC-MS is the time taken to achieve chromatographic separation of metabolites and to cool the system to a sufficiently low and stable temperature (30–40 °C) before introduction of the next sample. To speed sample throughput, various technologies aim to achieve faster GC cycle times (Fast-GC) or to remove the GC from the system entirely and use the mass spectrometer alone for metabolite analysis and the fingerprinting and classification of experimental samples. In proton transfer reaction mass spectrometry (PTR-MS), headspace air surrounding the fruit is drawn directly into the instrument where organic volatiles are ionized by reaction with protonated (charged) water molecules generated in a hollow cathode source. The resulting protonated organic volatiles move through a drift region before analysis by a quadrupole mass spectrometer. The related technology, selected ion flow tube mass spectrometry (SIFT-MS), generates ionized volatiles by reaction with a range of reagent ions such as H_3_O^+^, NO^+^ and O_2_^+^ with greater opportunities for more selective ionization [36] and for the resolution of volatiles having the same molecular mass [37].

PTR-MS/SIFT-MS methods have the advantages of minimal sample handling and processing and so have potentially high sample throughput. However, as individual volatiles are not separated by chromatography, ions of the same mass but derived from different volatiles cannot normally be distinguished and the measurement of the concentrations of individual volatiles requires the identification of unique ions or combinations of ions for that volatile species. PTR-MS has emerged as an alternative technology to GC-MS for the real-time analysis of volatiles in air and the mapping of quantitative trail loci (QTL) for volatile flavor compounds in fruit. PTR-MS was applied to the mapping of QTL for fruit flavor volatiles in a “Fiesta” X “Discovery” apple population [38]. Putative QTL were detected for seven mass spectral ions most of which could be related to specific volatiles or volatile classes. Real-time monitoring of herbivore-induced volatiles emission from branches of hybrid Aspen trees (*Populus* x) was followed in the field over 20 h of feeding by larvae of the autumn moth *Epirrita autumnata*. Emissions of green leaf volatiles occurred as sporadic peaks indicative of insect feeding while emissions of mono- and sesquiterpenes did not increase until 16 h after insect feeding began [12].

As part of a trend to study volatiles in the field, ion mobility mass spectrometry (IMS), where ions move in an electric field against a gas flow, offers high sensitivity for headspace volatiles in a portable instrument. IMS has been used to detect volatiles emitted by rotting samples of pine wood and to differentiate among the fungal species involved [39]. Differential mobility mass spectrometry, a variant of ion mobility mass spectrometry which uses the dependence of ion mobility on the strength of an applied electric field, has been coupled with thermal desorption of human breath volatiles and used to distinguish patients with chronic obstructive pulmonary disease from a control group of smokers who did not have this disease [40].

#### 2.3.2. HPLC/LC-MS Methods

High-performance liquid chromatography (HPLC) has been used for targeted profiling of specific volatile classes, aldehyde lipid oxidation products and amines, for example, which readily undergo selective chemical derivatization to form UV-absorbing or florescent chemical derivatives [41]. The advent of liquid chromatography coupled to mass spectrometry (LC-MS) offers new possibilities in the analysis of volatile biosynthesis and the direct analysis of the pools of non-volatile flavor precursors that are frequently present in biological systems often as glycoside, glucuronide, sulfate or phosphate derivatives. Such precursors provide a resource of volatiles that may released by plants in response to challenge or ingestion by herbivores. As these metabolites are often highly hydrophilic, and usually non-UV absorbing, they are difficult to analyze directly by HPLC. Traditionally, the identity of these metabolites has been inferred after isolation on XAD resins and hydrolysis with acid or glycosidase enzymes and subsequent GC-MS analysis. The availability of LC-MS should enable routine analysis of the volatile precursors as part of a metabolomics analysis of volatile biosynthesis and regulation in biological systems. Coupling SPME sampling with LC-MS may also allow direct *in vivo* sampling and measurement of these compounds both in plant and animal systems [23].

#### 2.3.3. Nuclear Magnetic Resonance Spectroscopy (NMR)

Nuclear magnetic resonance (NMR) has not been widely used in volatile analysis except for the analysis of essential oils but potentially offers quantitative analysis of multiple volatile metabolites with minimal sample processing [42-44]. NMR is a relatively insensitive technique requiring larger amounts of sample however this may be desirable when samples show high variability.

### 2.4. Data Analysis and Visualization Methods

Volatile profiling has a long history of application to biological problems, in particular to the sensory analysis and optimization of food storage conditions, where the emphasis has been on identifying biologically important metabolites (aroma impact compounds) or markers of food quality or degradation. There is a corresponding history of use of complex and multivariate statistical analysis to understand complex biological interactions analogous to current practice in metabolomics. For example, partial least squares regression (PLSR) was used to relate six sensory attributes of cherry wine to the concentrations of 51 odor active volatiles detected by gas chromatography-olfactometry, and measured and identified by GC-MS [45]. Similarly, principal components analysis (PCA) and PLSR were used to investigate the biochemical basis for the loss of capability for volatile production of ‘Fuji’ apples under three different controlled atmosphere (CA) storage conditions [46]. Differences in pyruvate decarboxylase activity were responsible for volatile differences between the two CA treatments, probably by providing substrate for alcohol acyltransferase catalyzed ester formation and suggesting that substrate availability is more important than enzyme activity for determining volatile production.

## 3. Applications of Volatile Analysis

### 3.1. Volatiles in Plant Biology

The role of volatiles in the internal and external communication of plants has been the subject of intense interest and is discussed in a number of excellent recent reviews [2,4-11] including the function of terpenes [47] and the complexity of plant volatile responses to multiple environmental stressors [5]. Plants emit volatiles from their roots, leaves, fruits and flowers and use these compounds internally as defensive and signaling systems to modulate levels of systemic acquired resistance (SAR) to pests and diseases and to alleviate abiotic stress. Volatiles also function as external signaling molecules (semiochemicals) contributing to the attractiveness of fruit and flowers to pollinators and to seed dispersers, respectively. They also contribute to the attraction of insect pests and beneficial insect predators in tritrophic interactions. For example, Lepidoptera larvae preferentially feed on the protruding glandular trichomes of tobacco leaves (*Nicotiana attenuata*) despite the defensive value of these trichomes which contain the insecticide nicotine. Ingestion of the *O*-acyl sugars found in these trichomes results in the rapid release by the feeding larvae of branched chain fatty acids which are attractants to a specific ground hunting predator ant [48].

Plant metabolism has traditionally been divided into primary and secondary metabolism with plant volatiles largely being considered products of secondary metabolism. Plant volatiles also include the phytohormones, ethylene, and methyl salicylate and jasmonate which, together with the fungicidal [49] green leaf aldehydes, 3-*Z*-hexenal and 2-*Z*-hexenal, and 3-*Z*-hexenyl acetate, and jasmonic acid, act to induce systemic acquired resistance to pests and diseases at remote sites both within and between neighboring plants [4,6]. Volatiles, including methyl jasmonate, may also act as alleopathic toxins to inhibit the growth of other plants. Monoterpenes such as α-pinene, camphene, and 1,8-cineol are emitted by many plants and have been shown to inhibit seed germination and early root growth across multiple plant species [50,51].

The importance of terpenes to plant defenses has been demonstrated in a number of studies. In some cases and for specialized pests or pathogens, plant volatiles may be required for normal plant-pest interactions (susceptibility) to occur. The importance of the monoterpene limonene (Figure 1) for the chemical defense of orange fruit peel (*Citrus sinensis*) has been demonstrated using fruit down-regulated for the synthesis of limonene which normally makes up to 97% of the total peel volatiles. Such knockdown fruit contained reduced amounts of limonene but instead of susceptibility showed resistance to both a specialized fungal pathogen of citrus *Penicillium digitatum* and to the bacterial pathogen *Xanthomonas citri* subsp. *citri*. Knockdown fruit were also less attractive to the males of the citrus pest medfly *Ceratitis capitata*, indicating the complex role of limonene in ecological interactions with fungal, bacterial and insect pests [52].

Volatiles may also influence food choice by mammalian herbivores resulting in differences in nutrient intake and effects on the sustainability and species diversity of the grazed plant ecosystems. The browsing by sheep on juniper *Juniperus communis* is negatively correlated with essential oil content and is associated with chemotypes of juniper dominated by the presence of the monoterpenes α-pinene and sabinene, rather than Δ-3-carene [53].

### 3.2. Improving Flavor of Foods

The flavor of many modern fruit and vegetable cultivars is widely recognized by consumers as being inferior to that of older cultivars [54]. The challenges of breeding for improved flavor while also selecting for the desirable traits of fruit firmness and good postharvest storage behavior has been discussed by Klee [54]. Flavor involves our perception of sugars, organic acids and of a diverse group of volatile metabolites produced by multiple pathways of primary and secondary metabolism. The pathways for volatile biosynthesis have only recently being determined [1]. They include biosynthesis from branched [55] and aromatic amino acids [56], production from fatty acids by hydroperoxidation (oxylipin) [57] and β-oxidation [58], biosynthesis of mono- and sesquiterpenes via the 2-*C*-methyl-D-erythritol-4-phosphate (MEP) and mevalonic acid (MVA) pathways [59], and the production of norisoprenoids such as α- and β-ionone by enzymatic oxidative cleavage of carotenoids [10,54,60]. Understanding the changes that occur in the regulation of this complex network of biosynthetic pathways as fruit and vegetables mature is an appropriate challenge for metabolomics.

Metabolomics approaches to the analysis of fruit volatiles have been applied in diverse fruits such as raspberry [61], strawberry [62], melon [63] and apple [20,64-66]. For tomato, there is an extensive literature on volatile concentrations in fruit of diverse genotypes [67,68] and related species [69], as well as an analysis of the role of volatile precursors in determining volatile availability in ripe fruit [70]. The integration of metabolomics with transcriptomics [71] and the metabolic profiling by GC-MS of interspecific introgression lines has enabled the identification of traits with potential for tomato improvement [72] and of loci which influence of the flavor of ripe fruit [73].

The aroma of the desirable aromatic Basmati and Jasmine rice varieties is related to the presence of 2-acetyl-1-pyrolline (2-AP). The presence of 2-AP has been mapped to a non-functional allele of the betaine aldehyde dehydrogenase (BADH2) gene on rice chromosome 8 [74].

### 3.3. Volatiles as Ecological Signals

Signaling using volatile organic compounds is a major means of communication among insects and other arthropods. The use of species specific volatiles by female insects to attract mates (pheromones) is well known but insects also use volatiles as group signals, as aggregation pheromones, to mark pathways between nest and food, and to mobilize for defense. Pherobase [75] is a comprehensive database of insect pheromones and semiochemicals containing entries on around 8,000 molecules including retention indexes, and mass spectral and NMR data for more than 2,500 compounds.

Volatiles also play an emerging role in the ecological interactions of fungi and microbes. These include novel metabolites such as the hydrocarbon sodorifen (C_16_H_26_), a bicyclic polymethylated diene of unknown biosynthetic origin (Figure 1) which is released by the root associated bacterium (rhizobacteria) *Serratia odorifera* [76]. Volatiles released by rhizobacteria may either inhibit or enhance plant growth as well as the growth of other microorganisms. Two out of six strains of plant-growth promoting rhizobacteria were found to release acetoin and 2,3-butanediol which promoted the growth of *Arabidopsis thaliana* seedlings [77]. Root inoculation with three species of plant growth-promoting rhizobacteria (*Pseudomonas fluorescens*, *Bacillus subtilis*, *Azospirillum brasilense*) also increased growth and the essential oil content of the herb *Oregano* x *majoricum* [78]. In more complex interactions, antagonistic strains of *Pseudomonas fluorescens and Serratia plymuthi* suppressed Agrobacterium crown gall tumors on tomato seedlings [79]. Evidence was presented for the involvement of volatiles and, in particular, of dimethyl disulfide in these interactions. Interestingly dimethyl disulfide (and ammonia) released by *Serratia odorifera* were implicated in inhibiting growth of *Arabidopsis thaliana* seedlings [80].

Some 250 volatiles produced by 129 fungal species have been compiled in a review by Chiron and Michelot [14] along with available evidence for their involvement in animal-fungal interactions. In addition to the usual array of hydrocarbons, alcohols, aldehydes and ketones, the fungal volatiles reported include unusual mono- and sesquiterpenes, heterocyclic compounds including indoles, pyrazines and an acetylene substituted thiophene, and also various lactones and complex polysulfides. Volatile production by fungi may also be used to signal toxicity to potential grazers. Thus Staaden *et al.* [81] showed that springtails (Collembola) used volatile cues to discriminate between fungi of different phylogenetic affiliations and to select a mutant strain with reduced secondary metabolite production. While many studies imply a role for secondary metabolites in deterring animals and in resisting host defenses, strong evidence is often lacking [82].

A number of frequently occurring volatiles (hexanal, 2-*E*-hexenal, 3-*Z*-hexenal) show antifungal activity [49] and have been developed as GRAS fumigants [83] and alternatives to synthetic chemicals. Hexanal shows activity against a number of fungi and is rapidly metabolized by apple fruit to naturally occurring volatile compounds [84].

### 3.4. Volatiles in the Environment

Volatile analysis provides a non-invasive tool to monitor environmental interactions in spatially complex or poorly accessible environments such as forests or microbial or marine communities and to track global cycling of chemical elements. Isoprene (C_5_H_8_, the simplest terpenoid) is emitted in vast quantities by land plants (trees) and provides protection from heat, oxidative and other abiotic stresses and repels herbivores [2,7,8]. Isoprene is the most important volatile hydrocarbon released by plants into the atmosphere with implications for global ozone levels and is responsible for the formation of organic aerosols (blue mountain haze) [85,86].

Biomethylation of sulfur, selenium, arsenic, and other metalloid elements by microorganisms or plants allows their volatilization and redistribution in the environment [87]. The volatile sulfur compounds, dimethylsulfide (CH_3_SSCH_3_) and methanethiol (CH_3_SH), are emitted by plants, algae and bacteria, especially in marine environments, and play a major role in the global sulfur cycle. These volatiles arise through the intermediacy of dimethylsulfonioproprionic acid (DMSP) (Figure 1) which occurs widely as an osmoprotective agent in algae and bacteria [88]. Lysis of DMSP by marine bacteria yields dimethylsulfide, while demethylation and subsequent lysis yields methanethiol [89].

Some plants also produce the methylated selenium derivatives dimethylselenium (CH_3_SeCH_3_), CH_3_SeSCH_3_ and dimethyl diselenium (CH_3_SeSeCH_3_). These volatiles may be useful tools for the phytoremediation of contaminated soils where metallic, metalloid or organic contaminants are concentrated into plant tissues, and may be removed from the site either by removal of the plant material or spontaneously by their volatilization as volatile metabolites. Tolerance to high-selenium soils in Se-hyperaccumulating plant species is related to the ability to synthesize methylselenocysteine and results in the emission of CH_3_SeCH_3_, CH_3_SeSCH_3_ and CH_3_SeSeCH_3_ from these plants [13]. Genetic modification of the non Se tolerant plant tobacco *Nicotiana tabacum* with a selenocysteine methyltransferase transgene caused a two to four-fold increase in Se accumulation, production of methylselenocysteine and generation of the volatiles CH_3_SeSCH_3_ and dimethyl diselenide when plants were watered with sodium selenate [90]. Arsenic (As) is also widespread in the environment and has multiple biotransformation pathways in microbes and algae. Methylation and volatilization to possibly less toxic compounds such as methyl- and dimethylarsenate, trimethylarsine oxide ((CH_3_)_3_AsO) and trimethylarsine ((CH_3_)_2_AsH) are thought to be important pathways in aquatic and soil environments [91].

Global cycling of the halogen elements, chlorine, bromine and iodine, by marine ecosystems includes major emissions of haloforms (CHCl_3_, CHBr_3_) and of other halogenated hydrocarbons (CH_2_Br_2_, CH_3_I). Volatiles produced by marine organisms also include unique polyhalogenated terpenes, phenols (Figure 1) and heterocycles (indoles) [32,92] produced through the action of vanadium-containing haloperoxidases. Fluorine is also incorporated into marine volatiles probably through a fluorinase enzyme operating via a S_N_2 type mechanism [93].

### 3.5. Humans and Volatiles

Volatiles (flavorings, aromas, scents) greatly affect our appreciation of our environment and the palatability and perceived quality of the foods we eat. Not all volatiles are readily or equally perceived as scented by humans, and large differences occur in odor threshold values recorded among individual humans [3]. Some volatiles of especially low sensory threshold (impact volatiles) may impart unique flavor notes to foods. Thus 2-methoxypyrazines such as 2-methoxy-3-isobutyl-pyrazine are produced in grapes and, at appropriate part per trillion concentrations (12–26 ng/L), contribute desirable vegetative or herbaceous flavor notes to Sauvignon blanc wines [94]. Volatiles also contribute to taints and off-flavors in foods. The sesquiterpene geosmin (Figure 1) is produced by several classes of microorganisms including Streptomyces and cyanobacteria (blue green algae) [95] and at low part per trillion concentrations is responsible for the unpleasant earthy taste of drinking water and, in combination with 2-methylisoborneol [96], for the flavor taint found in some freshwater fish. Instrumental and sensory assessment of food volatiles is widely used to evaluate food quality or the presence of pest and microbial infestations.

Volatiles produced by humans are of increasing interest as they reflect the different metabolic phenotypes (metabotypes) of individuals and may be useful as non-invasive biomarkers to evaluate and monitor disease or ‘health status’ [97]. Differences between the volatile profiles of individual humans (breath odor and taste of urine) are long known and have been used diagnostically as indicators of disease. Volatiles are also emitted by humans through the skin and in saliva [26], and are excreted in the faeces and urine [27] as a result of complex interactions between human metabolism and the intestinal microflora. Exhaled breath contains a complex mixture of volatiles, is easily obtained, and has been used as a marker of exposure of humans to occupational and environmental pollutants [98] and to distinguish patients with chronic obstructive pulmonary disease from a control group of healthy smokers [40]. Metabolic profiling by SPME of breath samples collected from allergic asthma and control children, led to the identification of 28 volatiles (mainly alkanes and aldehyde metabolites of oxidative stress) which allowed discrimination of asthmatic from control children with a classification rate of 88% [99]. A large number of the volatile compounds identified in saliva from 175 human subjects were identified as in common with compounds previously analyzed in sweat from the same subject group [26] but no statistical analysis of these relationships appears to have been presented.

## 4. Future Trends in Volatile Research

The range of ecological interactions mediated by volatiles seems limited more by experimental technique and historical precedent. Volatile interactions in the soil (plant rhizosphere) remain to be explored but are likely to be limited by diffusion rates to short range interactions. However the sesquiterpene α-caryophyllene, released from maize roots on insect feeding, is able to diffuse sufficiently to attract an insect-feeding nematode [47]. Ecological interactions in marine environments also involve ‘volatile’ compounds but remain relatively unexplored.

New methodology will meet some of these challenges. Improved high resolution GC-MS instruments will become more readily available allowing more selective detection and easier identification of metabolites. A move towards real-time analysis in environmental and field studies will see increased use of FAST-GC and of PTR-MS/IMS technologies which may be more portable and use the mass spectrometer to compensate for the omission of any chromatographic separation of individual volatiles. Newer DESI/DART technologies, which use an ion beam to extract metabolites directly from a sample in air into the mass spectrometer [100,101], will allow sampling and imaging of volatiles and volatile precursor with minimal processing of the biological sample. The cellular and sub-cellular distributions of volatiles and their precursors in plant organs are largely unknown and would not seem readily analyzable by other methods.

The availability of LC-MS offers the incorporation of volatile precursor analysis into future biological experiments, enabling a better understanding of volatile biosynthetic pathways and their responses to environmental challenge. Measurement throughout these pathways will allow integration of volatile responses with changes in gene expression, protein levels and enzymatic activities. As the role of volatiles in plant performance is increasingly recognized, volatile profiling will be integrated into metabolomics studies and used in combination with proteomic and transcriptomic analyses for improvement of commercially important plants. This will include mapping of QTL for volatiles for inclusion in molecular breeding.

The use of volatiles as natural biocontrol agents to manipulate the ecological interactions of pests and pathogens will provide continuing areas of research both in the field and in postharvest and food storage environments. Volatile analysis will continue to be applied for the non-invasive identification of health-related biomarkers in humans. New methodology including improved SPME [23,28] will see the intensive examination of human serum and urine for volatile biomarkers.
